# Cisplatin and ultra-violet-C synergistically down-regulate receptor tyrosine kinases in human colorectal cancer cells

**DOI:** 10.1186/1476-4598-11-45

**Published:** 2012-07-12

**Authors:** Junji Kawaguchi, Seiji Adachi, Ichiro Yasuda, Takahiro Yamauchi, Masanori Nakashima, Tomohiko Ohno, Masahito Shimizu, Takashi Yoshioka, Masahiko Itani, Osamu Kozawa, Hisataka Moriwaki

**Affiliations:** 1 Departments of Gastroenterology, Gifu University Graduate School of Medicine, Gifu 501-1194, Japan; 2 Departments of Pharmacology, Gifu University Graduate School of Medicine, Gifu 501-1194, Japan; 3 1-1 Yanagido, Gifu 501-1194, Japan

**Keywords:** Cisplatin, UV-C, EGFR, HER2, Down-regulation, Cell growth inhibition

## Abstract

**Background:**

Platinum-containing anti-cancer drugs such as cisplatin are widely used for patients with various types of cancers, however, resistance to cisplatin is observed in some cases. Whereas we have recently reported that high dose UV-C (200 J/m²) induces colorectal cancer cell proliferation by desensitization of EGFR, which leads oncogenic signaling in these cells, in this study we investigated the combination effect of low dose cisplatin (10 μM) and low dose UV-C (10 J/m²) on cell growth and apoptosis in several human colorectal cancer cells, SW480, DLD-1, HT29 and HCT116.

**Results:**

The combination inhibited cell cycle and colony formation, while either cisplatin or UV-C alone had little effect. The combination also induced apoptosis in these cells. In addition, the combination caused the downregulation of EGFR and HER2. Moreover, UV-C alone caused the transient internalization of the EGFR, but with time EGFR recycled back to the cell surface, while cisplatin did not affect its localization. Surprisingly, the combination caused persistent internalization of the EGFR, which results in the lasting downregulation of the EGFR.

**Conclusions:**

The combination of low dose cisplatin and low dose UV-C synergistically exerted anti-cancer effect by down-regulating RTK, such as EGFR and HER2. These findings may provide a novel strategy for the treatment of patients with colorectal cancer.

## Introduction

Among the receptor tyrosine kinases (RTKs), the ErbB family, such as epidermal growth factor receptor (EGFR; ErbB1) or human epidermal growth factor receptor-2 (HER2; ErbB2) plays a pivotal role in regulating a number of cellular processes including cell proliferation, survival and migration [1], and dysregulation of EGFR activity leads to tumorgenesis [2]. Mechanisms leading to oncogenic signaling behind EGFR are thought as follows: 1) increased EGFR levels, 2) autocrine and/or paracrine growth factor loops, 3) heterodimerization with other EGFR family members and cross-talk with heterologous receptor systems, 4) defective receptor downregulation, and 5) activating mutations [3].

We have previously reported that the blockade of EGF stimulation significantly suppressed colorectal cancer cell growth, suggesting that the EGFR pathway plays an important role in proliferation of these cells [4]. Thus, EGFR downregulation is a critical target for therapy against colorectal cancer that is highly dependent on EGFR. As for HER2, their expression has been first reported to be amplified in breast cancer [5]. Since clinical and experimental evidences show a role for over-expression of the HER2 protein in the progression of human breast, ovarian, non-small cell lung [6] and colorectal cancer [7], HER2 may be a candidate target for receptor-targeted therapeutics.

Cis-diamminedichloroplatinum (CDDP) or cisplatin is one of the most effective DNA-damaging anti-tumor agent and is used for the treatment of various human cancers [8-10]. However, resistance to cisplatin arises in some cases and many compounds combined with platinum-based drugs are now ongoing clinical trials [11]. Increasing evidences show that cisplatin directly influences EGFR signaling. Cisplatin reportedly induces EGFR internalization [12], phosphorylation at Thr1045 mediated via a ubiquitin ligase, c-Cbl [13] and phosphorylation at Thr669, at a site which is phosphorylated by p38 MAPK [14], while activation of stress-activated protein kinase/*c-Jun*-N-terminal kinase or p38 MAPK by cisplatin has been reported to promote apoptotic cell death [15]. In addition, in many studies researchers have used cisplatin at relatively higher doses (30 μM or more), which is impractical *in vivo*.

Ultra-violet (UV) radiation is divided into three bands: UV-A (320–400 nm), UV-B (280–320 nm) and UV-C (200–280 nm). Most of the UV-C and approximately 90% of UV-B are absorbed while passing through the atmospheric layers. UV-A and UV-B are recognized harmful for humans, while UV-C is used for studying DNA damage and cellular DNA repair process [16]. So far, the possibility of application rather for treatment of human cancer has been demonstrated [17,18]. In a series of papers, Petersen et al have investigated the photophysical consequences of illuminating the aromatic residues of proteins with UV-C [19-25]. In particular, they demonstrated that 280 nm UV illumination of aromatic residues in proteins causes the disruption of nearby disulphide bridges, where EGFR are excessively populated, leading to the suppression of the proliferative potential in human cancer cell lines [17].

Whereas we recently reported the availability of UV-C alone (30 J/m² and more) in human colorectal cancer cells, in which we showed that UV-C can evade these cells from oncogenic stimulation of EGF by decreasing the EGFR protein level [26], we herein investigated the combination use of low dose cisplatin and low dose UV-C on cell growth in human colorectal cancer cells (SW480, HT29, DLD-1 and HCT116) and found that the combination has synergistic effect on cell growth inhibition by down-regulating receptor tyrosine kinases, such as EGFR and HER2.

## Results

### Effects of cisplatin and/or UV-C on cell proliferation in human colorectal cancer cells

**Figure 1:**
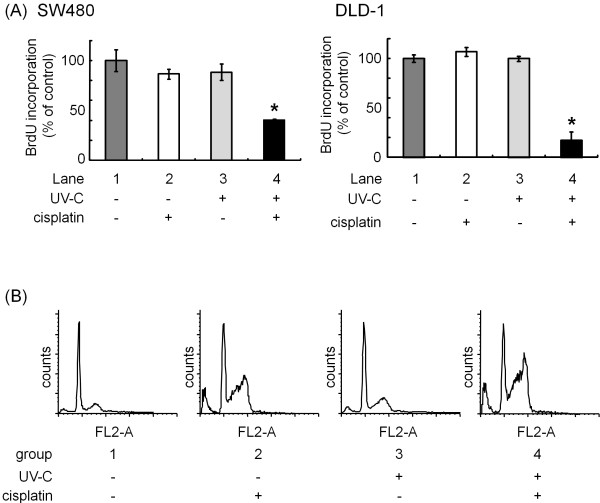
** (A) Effects of cisplatin and/or UV-C on cell proliferation in human colorectal cancer cells.** SW480 and DLD-1 cells were either exposed to 10 J/m² UV-C (lanes 3), treated with 10 μM cisplatin (lanes 2), or received both (lanes 4). Twenty four h later, the measurement of BrdU incorporation was performed using cell proliferation ELISA (BrdU). Results are expressed as percentage of incorporation with 100% representing that by untreated control cells (lanes 1). (**B**) SW480 cells were treated with 10 μM cisplatin (group 2), 10 J/m² UV-C (group 3) or combination of 10 μM cisplatin and 10 J/m² UV-C (group 4). The cells were then stained with propidium iodide (PI) to analyze progression of cell cycle. The distribution of cells in the apoptosis and each phase of cell cycle were calculated in each group. Bars designate SD of triplicate assay. The asterisks (*) indicate significant decrease (p < 0.05) as compared to the corresponding control, respectively.

**Figure 2:**
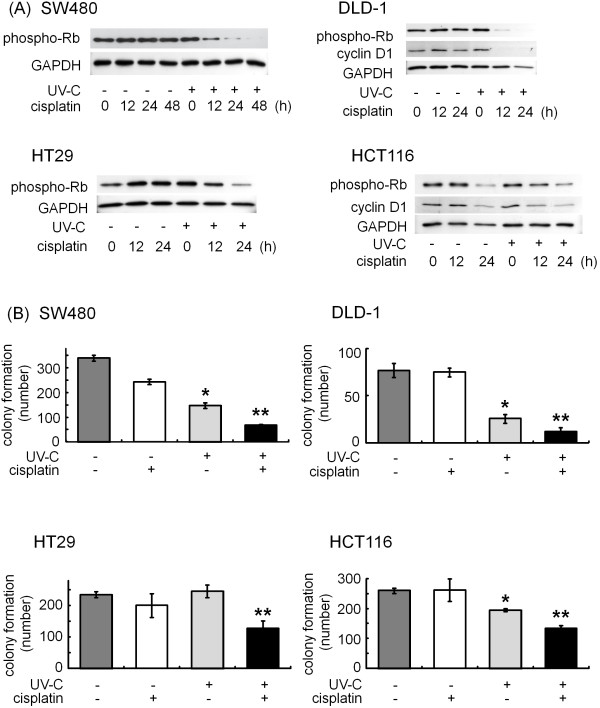
** (A) Effects of cisplatin and/or UV-C on cell proliferation markers, phospho-Rb and cyclin D1 in human colorectal cancer cells.** SW480, DLD-1, HT29 and HCT116 cells were first exposed to 10 J/m² of UV-C or not, and then treated with 10 μM of cisplatin for the indicated periods. Protein extracts were harvested and examine by Western blotting using anti-phospho-Rb and anti-cyclin D1 antibodies. (**B**) Effects of cisplatin and/or UV-C on colony formation in SW480, DLD-1, HT29 and HCT116 human colorectal cancer cells. The attached human colorectal cancer cells were first exposed to the indicated doses UV-C (0 or 10 J/m²), just after the aspiration of the growth medium. The cells were then incubated in normal growth media with/without 10 μM cisplatin for 24 h. After trypsinization, the counted cells (3 x 10^3^) were re-seeded into new culture dishes and incubated for 7 days. The cells were then fixed with clonogenic reagent (see Materials and methods) and the average number of colony from 5 randomly chosen fields (x 20) were counted, respectively. Bars designate SD of triplicate assay. The asterisks (* and **) indicate significant decrease (p < 0.05) as compared to the control and UV-C alone, respectively.

Bromodeoxyuridine (BrdU) is a synthetic thymidine analog that gets incorporated into DNA during cell division. Therefore, the measurement of BrdU incorporation reflects the ability of cell growth. We first investigated the effects of cisplatin and/or UV-C on cell proliferation using BrdU. Whereas either 10 μM of cisplatin or 10 J/m² of UV-C hardly affected BrdU incorporation in SW480 and DLD-1 cells (Figure 1A, lanes 2 and 3, respectively), the combination caused a marked inhibition in BrdU incorporation (Figure 1A, lane 4, respectively). While it has previously been reported that cisplatin induces cell cycle arrest at the G2-phase [27], cell cycle analysis using flow cytometry revealed that the combination of cisplatin and UV-C increased the population at G2/M phases (28.2 ± 1.35%), compared with cisplatin (21.9 ± 0.68%; p = 0.0014) or UV-C (15.2 ± 0.76%; p = 0.0004) (Figure 1B). Moreover, we examined the protein level of phospho-Rb and cyclin D1, both of which direct cells toward proliferation by controlling progression through the restriction point of cell cycle (Figure 2A) [28]. In SW480 cells, cisplatin by itself had little effect on phosphorylation level of Rb. However, when the cells were first exposed to UV-C and then incubated in the presence of cisplatin, the protein level of phospho-Rb was decreased in a time-dependent manner after 12 h (Figure 2). Since we have recently reported that 10 J/m² of UV-C did not cause the decrease in the protein level of Rb [26], these results suggest that the combination of cisplatin and UV-C exerts synergistic effect on the suppression of cell cycle. We also verified the combination effect in DLD-1, HT29 and HCT116, other human colorectal cancer cell lines (Figure 2).

### Effects of cisplatin and/or UV-C on colony formation in human colorectal cancer cells

We next performed colony formation assay, which is a microbiology technique for studying the effectiveness of specific agents on the survival and proliferation of cells (Figure 2B) [29]. The combination synergistically suppressed colony formation of SW480 cells, although cisplatin or UV-C alone did to a lesser extent. Similarly, the combination synergistically decreased the number of colony formation in DLD-1 and HCT116 cells, whereas UV-C alone slightly affected them in these cells. As for HT29 cells, while cisplatin or UV-C alone has no effect, the combination synergistically suppressed colony formation. As a whole, these results suggest that the combination has cytocidal effects on several colorectal cancer cells.

### Effects of cisplatin and/or UV-C on the apoptosis in human colorectal cancer cells

**Figure 3:**
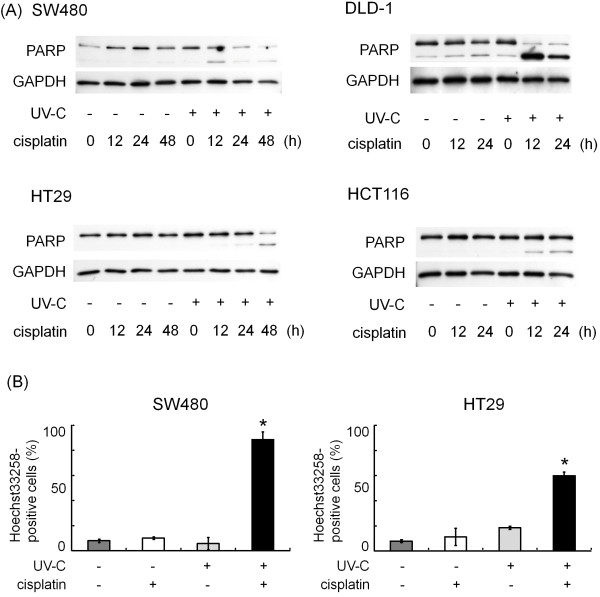
**(A) Effects of cisplatin and/or UV-C on PARP cleavage and DNA fragmentation in human colorectal cancer cells.** SW480, DLD-1, HT29 and HCT116 cells were first exposed to the indicated doses of UV-C (0 J/m² or 10 J/m²), and then treated with/without 10 μM of cisplatin for the indicated periods. Protein extracts were then harvested and examine by Western blotting using anti-PARP and anti-GAPDH antibodies. (**B**) SW480 and HT29 cells were first exposed to the indicated doses of UV-C (0 J/m² or 10 J/m²), and then treated with/without 10 μM of cisplatin for the indicated periods. They were then treated with Hoechst 33258 and were examined by fluorescence microscopy. The numbers of Hoechst33258-positive cells (apoptotic nuclei) from 5 randomly chosen fields (x 40) were counted, respectively. Bars designate SD of triplicate assay. The asterisks (*) indicate significant increase (p < 0.05) as compared to the corresponding controls, respectively.

We next investigated the combination effect of cisplatin and UV-C on apoptosis by observing PARP cleavage, since PARP is a family of proteins involved in a number of cellular processes involving mainly DNA repair and programmed cell death, indicating cell apoptosis [30]. While cisplatin or UV-C alone had little effect on PARP, the combination caused PARP cleavage in SW480, DLD-1, HT29 and HCT116 cells (Figure 3A). While Hoechst33258 are used to stain DNA and easily detect such DNA fragments, we next examined the effect of combination of cisplatin and UV-C on DNA fragmentation utilizing this dye and found that the combination increased the number of Hoechst 33258-positive apoptotic cells in SW480 and HT29 cells (Figure 3B), which are consistent with our results shown in Figure 3A.

### Effects of cisplatin and/or UV-C on the protein level of EGFR and HER2 in human colorectal cancer cells

**Figure 4:**
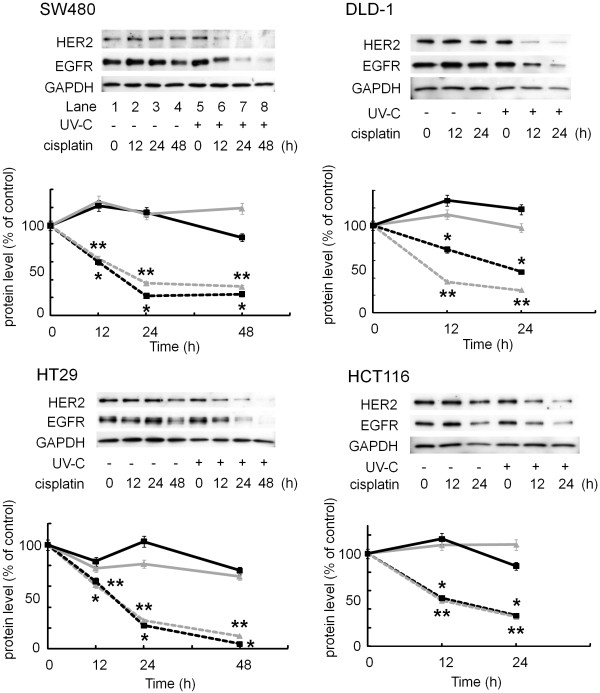
** Effects of cisplatin and/or UV-C on HER2 and EGFR in human colorectal cancer cells.** SW480, DLD-1, HT29 and HCT116 cells were first exposed to the indicated doses of UV-C (0 J/m² or 10 J/m²), and then treated with/without 10 μM of cisplatin for the indicated periods. Protein extracts were then harvested and examine by Western blotting using anti-HER2, anti-EGFR and anti-GAPDH antibodies. The lower line graphs show quantification data for the protein levels of HER2 and EGFR, after normalization to GAPDH, respectively. Bars designate SD of triplicate assay. The asterisks (* and **) indicate significant decrease (p < 0.05) as compared to the corresponding controls, respectively.

As described in Introduction, EGFR downregulation is the most prominent regulatory system in signal attenuation and involves the internalization and subsequent degradation of the activated receptor in the lysosomes. As well, HER2 is frequently overexpressed in colorectal cancer when compared with normal colonic mucosa, and the extent of overexpression seems to correlate with increasing disease stage and poorer patient survival [31]. Therefore, therapies that target the EGFR and/or HER2 may be effective in the chemoprevention and/or therapy of colorectal cancer [32]. Whereas we recently reported that EGFR signaling plays a critical role in proliferation of colorectal cancer cells [26], we next focused on the expression level of EGFR as well as HER2 in several colorectal cancer cells including SW480, DLD-1, HT29 and HCT116, since we observed that the combination use of cisplatin and UV-C synergistically exerts suppressive effect on cell proliferation and apoptosis (Figures 1 and 3). As depicted in Figure 4, 10 μM cisplatin alone did not affect these levels even after a longer treatment in SW480 (Figure 4, lanes 1–4). As well, while UV-C at a dose over 30 J/m² caused a marked decrease in the EGFR protein level [26], in this study we observed that 10 J/m² of UV-C did not affect (Additional file 1). Interestingly, the combination use of 10 μM cisplatin and 10 J/m² UV-C clearly induced the decrease in the protein levels of EGFR as well as HER2 in SW480 cells, which were appeared at 12 h after treatment with cisplatin and UV-C (Figure 4, lanes 5–8). Similar results were observed in other colorectal cancer cells, DLD-1, HT29 and HCT116. Together, the combination effect of cisplatin and UV-C on the suppression of cell growth seems to be due to the down-regulation of EGFR and/or HER2.

### Effects of cisplatin and/or UV-C on the internalization of EGFR in SW480 cells

**Figure 5:**
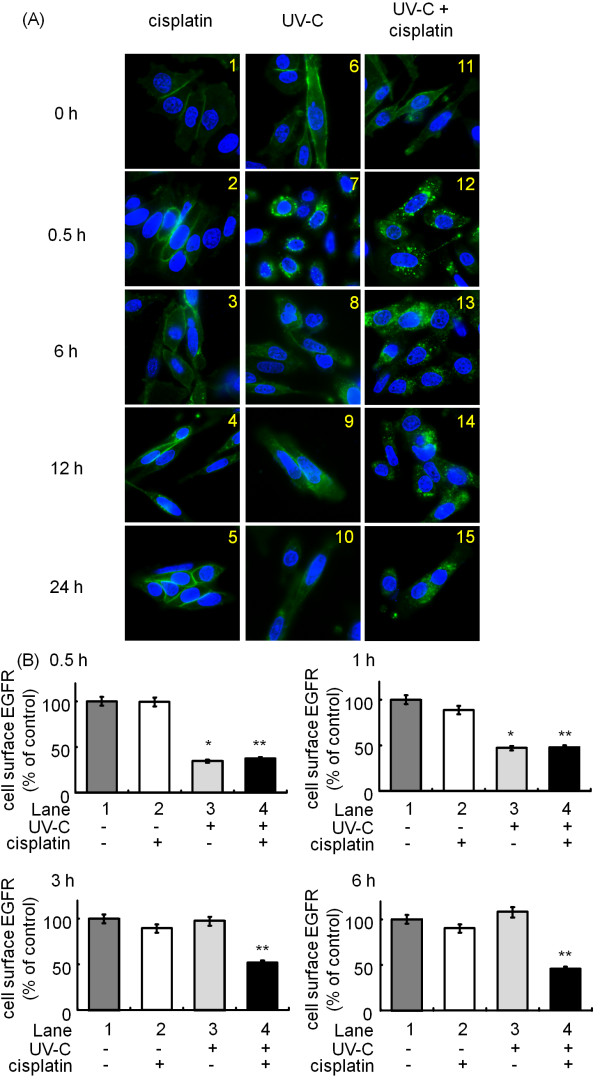
** (A) Effects of cisplatin and/or UV-C on the localization of EGFR in SW480 cells.** SW480 cells were first labeled for 15 min at 37°C with anti-EGFR antibodies. They were then exposed to 10 J/m² of UV-C (panels 6–10 and 11–15, respectively) or not (panels 1–5, respectively), followed by the treatment with (panels 1–5 and 11–15, respectively) or without (panels 6–10, respectively) 10 μM of cisplatin for the indicated periods at 37°C. After fixation and permeabilization, the cells were stained with Alexa 488® conjugated anti-mouse secondary antibody for EGFR (green signal) and DAPI (blue signal) for 1 h, and then examined by fluorescence microscope. (**B**) Effects of cisplatin and/or UV-C on the amount of cell surface EGFR in SW480 cells. SW480 cells were first labeled for 15 min at 37°C with an anti-EGFR antibody that recognizes the extracellular domain of the EGFR. They were then exposed to 10 J/m² UV-C (lanes 3 and 4) or not (lanes 1 and 2), followed by the treatment with (lanes 2 and 4) or without 10 μM cisplatin (lanes 1 and 3) for the indicated periods at 37°C. The amount of cell surface EGFR was then measured by ELISA. The asterisks (* and **) indicate significant decrease (p < 0.05) with respect to the control (lane 1, respectively). For additional details see Materials and methods.

It has previously been reported that UV irradiation (100 J/m²) induces rapid and persistent internalization of EGFR [33]. As well, we have recently reported that UV-C at a dose over 30 J/m² caused the internalization and subsequent down-regulation of the EGFR in SW480 cells [26]. In order to elucidate the mechanism underlying combination effect of cisplatin and UV-C, we next examined whether cisplatin (10 μM) and/or UV-C (10 J/m²) induces changes in the subcellular localization of EGFR in SW480 cells. Whereas antibody-tagged EGFR remained on the cell surface (Figure 5A, panels 1, 6 and 11), 0.5 h incubation after the treatment of the cells with UV-C alone (10 J/m²) resulted in the distribution of the EGFR to cytosol beneath the plasma membrane, thus indicating that UV-C indeed induced the internalization of the EGFR (Figure 5A, panel 7). By contrast, cisplatin (10 μM) by itself did not affect the localization of the EGFR (Figure 5A, panels 2–5). Interestingly, when the cells were first exposed to UV-C and then incubated in the absence of cisplatin for 6 h and more, the antibody-tagged EGFR reappeared on the cell surface, thus suggesting that internalized EGFR recycled back to the cell membrane (Figure 5A, panels 8–10). However, the EGFR remained to be internalized when the cells were treated with the combination of cisplatin and UV-C (Figure 5A, panels 12–15).

To verify these results, we measured the amount of cell surface EGFR by enzyme-linked immunosorbent assay (ELISA). Whereas UV-C alone decreased the amount of cell surface EGFR within 0.5 h (Figure 5B, lane 3). However, they were gradually recovered 3 h after treatment with UV-C (Figure 5B, lanes 3, respectively). On the contrary, cell surface EGFR in the cells treated with the combination of cisplatin and UV-C remained to be decreased (Figure 5B, lanes 4, respectively). Taken together with our results obtained from fluorescence study, we strongly suggest that the treatment with cisplatin after UV-C exposure blocks the recycling of the EGFR which are internalized by UV-C.

## Discussion

Platinum-containing anti-cancer drugs, including cisplatin, inhibit DNA replication [34,35] and RNA transcription [36], and induce cell cycle arrest at the G2-phase and apoptosis [27,37]. However, cisplatin at a higher dose concomitantly raises severe adverse effects, such as myelo-supression, nausea, anorexia, diarrhea and liver dysfunction. Therefore, many trials have made effort to minimize the dose of cisplatin in cancer patients. In the present study, we examined the combination effect of low dose cisplatin (10 μM) and low dose UV-C (10 J/m²) on human colorectal cancer cells, while we recently reported the potential availability of UV-C in these cells [26].

We herein demonstrated that the combination use synergistically inhibited the cell proliferation by BrdU assay (Figure 1A), flow cytometry (Figure 1B), Western blotting (Figure 2A) and colony formation assay (Figure 2B). We also unveiled that the cisplatin and UV-C have synergistic effect on apoptosis, while cisplatin or UV-C alone had little effect (Figure 3). They were accompanied by downregulation of RTKs, such as EGFR and HER2 (Figure 4), both of which reportedly play a critical role in cell proliferation in many types of cancers including colorectal cancer [7,38].

**Figure 6:**
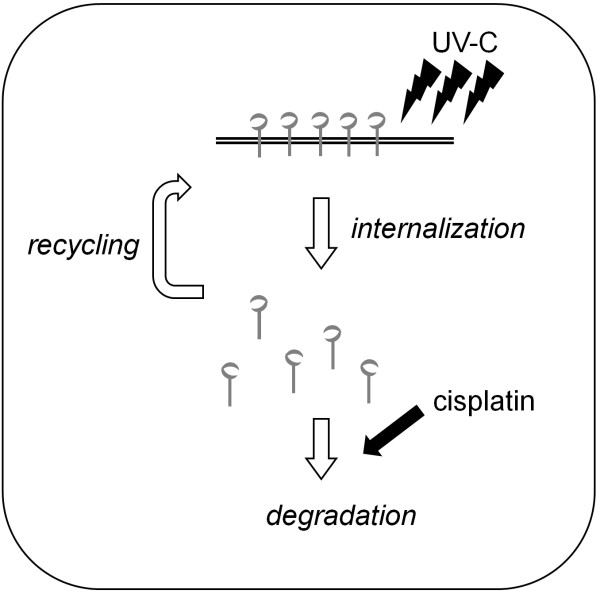
** Schematic representation of the combination effect of cisplatin and UV-C in human colorectal cancer cells**. After UV-C exposure even at a low dose, cell surface EGFR is internalized. With time the internalized EGFR by UV-C recycles back to the cell membrane, but cisplatin blocks this recycling and induces EGFR degradation, resulting in cell cycle arrest.

An anti-EGFR monoclonal antibody inhibits EGFR activation, resulting in the enhancement of the anti-cancer effect of cisplatin [39,40]. Indeed, chemotherapy with cetuximab or panitumumab, both of which are also anti-EGFR monoclonal antibodies, can prolong survival period of colorectal cancer patients by nearly twenty-four months [41-43]. On the contrary, it has recently been reported that EGFR inhibition can protect EGFR from cisplatin-mediated phosphorylation and subsequent ubiquitination and degradation, indicating that treatment with an EGFR inhibitor before cisplatin would be antagonistic [13]. Thus, the efficacy of the combination of cisplatin and EGFR targeting drugs remains to be elucidated. In this study, low dose UV-C (10 J/m²) induced EGFR internalization, but these receptors recycled back to the cell surface, whereas the combination use of cisplatin and UV-C induced persistent EGFR internalization (Figure 5). It has previously been reported that if cisplatin-bound EGFRs remain on the cell surface, they catalytically inhibit cell death [33]. Therefore, we speculate that pretreatment with UV-C helps cisplatin to induce degradation of EGFR, since UV-C alone caused EGFR internalization into the perinuclear area of the cells, where cisplatin might exert maximum effect on the donwregulation of EGFR (summarized in Figure 6). Nevertheless, further investigation is required to elucidate why UV-C causes EGFR internalization and why cisplatin induces EGFR degradation.

Regarding the mechanisms underlying EGFR down-regulation, they involve several important phosphorylation sites in EGFR, including Tyr1045, a docking site for the ubiquitin ligase c-Cbl, and Ser1046/1047, which are required for EGFR desensitization in EGF-treated cells [44,45]. We recently found that (−)-epigallocatechin-3-gallate as well as heat shock protein 90 inhibitors cause down-regulation of the EGFR via phosphorylation at Ser1046/1047 through p38 MAPK in human cancer cells [46,47]. However, we did not observe the phosphorylation of EGFR at these residues when the cells were treated with low dose cisplatin and/or low dose UV-C in colorectal cancer cells (data not shown). Therefore, it seems that EGFR degradation by the combination does not depend on Tyr1045 or Ser1046/1047. Moreover, it has previously reported that p38 MAPK plays an important role in 100 J/m² UV-induced EGFR internalization [33]. However in the present study, the combination did not influence the phosphorylation of p38 MAPK (data not shown). These results also suggest that the synergistic effect of cisplatin and UV-C also does not depend on p38 MAPK activation.

Initial platinum treatment is generally responsive, but the majority of cancer patients eventually relapse with cisplatin-resistance [10,48]. Several mechanisms of resistance to cisplatin are proposed; 1) reduced drug uptake, 2) increased drug inactivation, 3) increased DNA adduct repair, and 4) defective apoptotic response [10]. Importantly, a poor response of human cancers to cisplatin is associated with amplification and over-expression of HER2 found in some of breast and ovarian cancer patients [10,48]. Since we showed that the combination use of cisplatin and UV-C down-regulated HER2 (Figure 4), UV-C could alter the resistance to cisplatin in human colorectal cancer cells.

## Conclusions

These results suggest that UV-C synergizes with cisplatin in the downregulation of receptor tyrosine kinases in human colorectal cancer cells. Our findings could provide a new aspect for the treatment of patients with colorectal cancer, although further investigation is required to develop devices that supply UV-C efficiently into human colorectal cancer, for example with endoscopic/laparoscopic approach.

## Materials and methods

### Materials

Antibodies against total EGFR and glyceraldehyde-3-phosphate dehydrogenase (GAPDH) were obtained from Santa Cruz Biotechnology (Santa Cruz, CA). Antibodies against total HER2, cyclin D1, phospho-retinoblastoma (Rb) and poly (ADP-ribose) polymerase (PARP) were purchased from Cell Signaling (Beverly, MA). Cisplatin was purchased from Sigma Aldrich (St. Louis, MO). ECL Western blot detection system was purchased from GE Healthcare (Buckinghamshire, UK). Cell Proliferation ELISA (BrdU) was obtained from Roche Diagnostics Co (Indianapolis, IN). Alexa Fluor 488® conjugated donkey anti-mouse IgG antibodies and 4',6-diamidino-2-phenylindole (DAPI) were purchased from Invitrogen and Wako (Tokyo, Japan), respectively. p2'-(4-Hydroxyphenyl)-5-(4-methyl-1-piperazinyl)-2, 5'-bi-*1 H*-benzimidazole, trihydrochloride (Hoechst 33258) solution was purchased from Dojindo (Kumamoto, Japan). PI/RNase Staining Buffer was obtained from Becton Dickinson (Franklin Lakes, NJ). Other materials and chemicals were obtained from commercial sources.

### Cell culture

SW480 and HT29 human colorectal cancer cells, that were obtained from American Type Culture Collection (Manassas, VA), were grown in Dulbecco's modified Eagle’s medium (DMEM) (Invitrogen, San Diego, CA), containing 10% fetal calf serum (FCS) with penicillin (100 U/ml) and streptomycin (100 μg/ml) in a humidified 5% CO_2_ incubator at 37°C. DLD-1 and HCT 116 human colorectal cancer cells were from American Type Culture Collection (Manassas, VA) and grown in Roswell Park Memorial Institute 1640 (RPMI) (Invitrogen, San Diego, CA) as described above.

### UV-C exposure

UV-C exposure of cells was performed in UV-C 500 UV Crosslinker (8 W 254 nm UV lamp) (GE Healthcare), which creates CW light using 8 W 254 nm UV lamps. Fluorescent lamps without a phosphorescent coating emit UV with two peaks at 254 nm and 185 nm due to the peak emission of the mercury within the bulb. UV lamps used quartz (glass) block the 185 nm wavelength and emit only 254 nm UV. After aspiration of the growth medium, the cells were exposed to the indicated dose (J/m² = 100 μJ/cm²) of UV-C in 5 sec, and then incubated for the indicated times.

### Cell proliferation assay

BrdU incorporation was measured using cell Proliferation ELISA (BrdU). The cells (7 x 10^3^/well) were seeded onto 96-well plates and 48 h later, the cells were exposed to the indicated doses (0 or 10 J/m²) of UV-C, just after the aspiration of the growth medium. The cells were then incubated in DMEM or RPMI medium with 1% FCS and 10 μM of cisplatin for 24 h. They were then used for the assay according to the manufacturer’s protocol. All assays were done at least three times.

### Cell cycle analysis

Cell cycle analysis was done as described previously [7]. In brief, SW480 cells were exposed to UV-C, followed by the incubation in DMEM with/without 10 μM of cisplatin for 96 h. The cells were then harvested and stained with 500 μl of PI/RNase staining buffer for 15 min at room temperature. They were finally analyzed by flow cytometry using a FACS Calibur instrument (Becton Dickinson); data were analyzed using the CELL Quest computer program (Becton Dickinson) as previously described. All data were obtained from at least three independent experiments.

### Colony formation assay

Human colorectal cancer cells (SW480, DLD-1, HT29 and HCT116) were exposed to UV-C and then incubated in DMEM or RPMI medium and with/without 10 μM of cisplatin. Twenty four h after treatment, the cells were trypsinized and the cells (3 x 10^3^) were re-seeded into fresh tissue culture dishes and incubated for 7 days. Fresh media were added at day 4. At day 7, the media were removed and the cells were fixed with 2 ml of clonogenic reagent (50% ethanol, 0.25% 1,9-dimethyl-methylene blue) for 45 min. They were then washed with PBS twice and counted the blue colonies on 5 randomly chosen fields.

### Western blotting

The cells were lysed in lysis buffer [20 mM Tris/HCl (pH 7.5), 150 mM NaCl, 1 mM EDTA, 1 mM EGTA, 1% TritonX-100, 2.5 mM sodium pyrophosphate, 50 mM NaF, 50 mM HEPES, 1 mM Na_3_VO_4_ and 2 mM phenylmethylsulfonyl fluoride (PMSF)] and scraped from the Petri dishes. Protein extracts were examined by Western blot analysis as previously described [49,50].

### Immunofluorescence microscopy studies

Immunofluorescence microscopy studies were performed as described previously [46]. Live cells grown on coverslip-bottom dishes in DMEM were first exposed to the mouse anti-EGFR antibody that recognized the extracellular domain of EGFR for 15 min and then exposed to UV-C (10 J/m²) and/or cisplatin (10 μM) and incubated in DMEM for the indicated times (0.5 h, 6 h, 12 h and 24 h) at 37°C. They were then fixed with 4% paraformaldehyde for 10 min on ice and then exposed to 0.1% Triton X-100 for 10 min to permeabilize the cell membrane. They were followed by exposure to Alexa Fluor 488® conjugated donkey anti-mouse IgG antibodies (green signal) and 4',6-diamidino-2-phenylindole (DAPI) for 1 h. The cells were then examined by fluorescence microscopy, BIOREVO (BZ-9000) (Keyence, Tokyo, Japan) according to the manufacturer’s protocol.

### Quantification of cell surface EGFR by enzyme-linked immunosorbent assay (ELISA)

Quantification of cell surface EGFR was performed as described previously [26]. In brief, SW480 cells were first exposed to the mouse anti-EGFR antibody in DMEM containing 1% BSA, for 15 min at 37°C. The cells were then incubated for the indicated times in DMEM with/without 10 μM of cisplatin after exposure to UV-C, then fixed with 4% paraformaldehyde for 10 min on ice. After blocking with 1% BSA in PBS for 1 h, the cells were exposed to an anti-mouse IgG, horseradish peroxidase-linked whole antibody (GE healthcare, Piscataway, NJ) for 1 h at room temperature, followed by washing four times with PBS containing 1% BSA. Finally, the cells were exposed to 50 μl of 1-stepTM Ultra TMB-ELISA reagent (Pierce, Rockford, IL) for 5 min at room temperature. The absorbance of each sample at 450 nm was then measured.

### Hoechst 33258 staining

Live cells grown on coverslip-bottom dishes were first exposed to UV-C (10 J/m²) and/or cisplatin (10 μM) for 72 h and then stained with Hoechst 33258 in DMEM without FCS for 1 h at 37°C. They were then fixed with 4% paraformaldehyde for 10 min on ice. The cells were then examined by fluorescence microscopy, as described above.

### Densitometric analysis

The densitometric analysis was performed using scanner and image analysis software (Image J ver. 1.45 g). The back ground subtracted signal intensity of each protein signal was normalized by the respective control signal. All data were obtained from at least three independent experiments.

### Statistical analysis

The data were analyzed by ANOVA followed by Bonferroni method for multiple comparisons between the indicated pairs, and a p < 0.05 was considered significant.

## Abbreviations

UV-C: Ultra-violet-C; RTKs: Receptor tyrosine kinases; EGF: Epidermal growth factor; EGFR: EGF receptor; HER2: Human epidermal growth factor receptor-2; GAPDH: Glyceraldehyde-3-phosphate dehydrogenase; Rb: Retinoblastoma protein; PARP: Poly (ADP-ribose) polymerase; MAPK: Mitogen-activated protein kinase; GSK: Glycogen synthase kinas; DMEM: Dulbecco's modified Eagle’s medium; RPMI: Roswell Park Memorial Institute 1640; FCS: Fetal calf serum; PBS: Phosphate buffered saline; BrdU: Bromodeoxyuridine (5-bromo-2'-deoxyuridine); ELISA: Enzyme-linked immunosorbent assay; DAPI: 4':6-diamidino-2-phenylindole; Hoechst 33258: p2'-(4-Hydroxyphenyl)-5-(4-methyl-1-piperazinyl)-2: 5'-bi-*1H*-benzimidazole: trihydrochloride.

## Competing interests

The authors declare that they have no competing interests.

## Authors’ contributions

SA designed the research studies; SA, JK, TY, MN, TO, MS, TY and MI carried out the molecular biological studies; SA, IY, OK and HM analyzed and interpreted the data; JK wrote the draft of the manuscript. All authors read and approved the final manuscript.

## Supplementary Material

Additional file 1 ** Effect of 10 J/m² UV-C on HER2, EGFR, phospho-Rb and cyclin D1 in human colorectal cancer cells.** SW480, DLD-1, HT29 and HCT116 cells were exposed to 10 J/m² of UV-C and then treated for the indicated periods. Protein extracts were then harvested and examine by Western blotting using anti-HER2, anti-EGFR, anti-phospho-Rb, anti-cyclin D1 and anti-GAPDH antibodies. Click here for file
