# Supplementary figures and images for: Cisplatin and ultra-violet-C synergistically down-regulate receptor tyrosine kinases in human colorectal cancer cells
Source: Mol Cancer. 2012 Jul 12;11:45. doi: 10.1186/1476-4598-11-45 (PMC3477093; doi:10.1186/1476-4598-11-45)

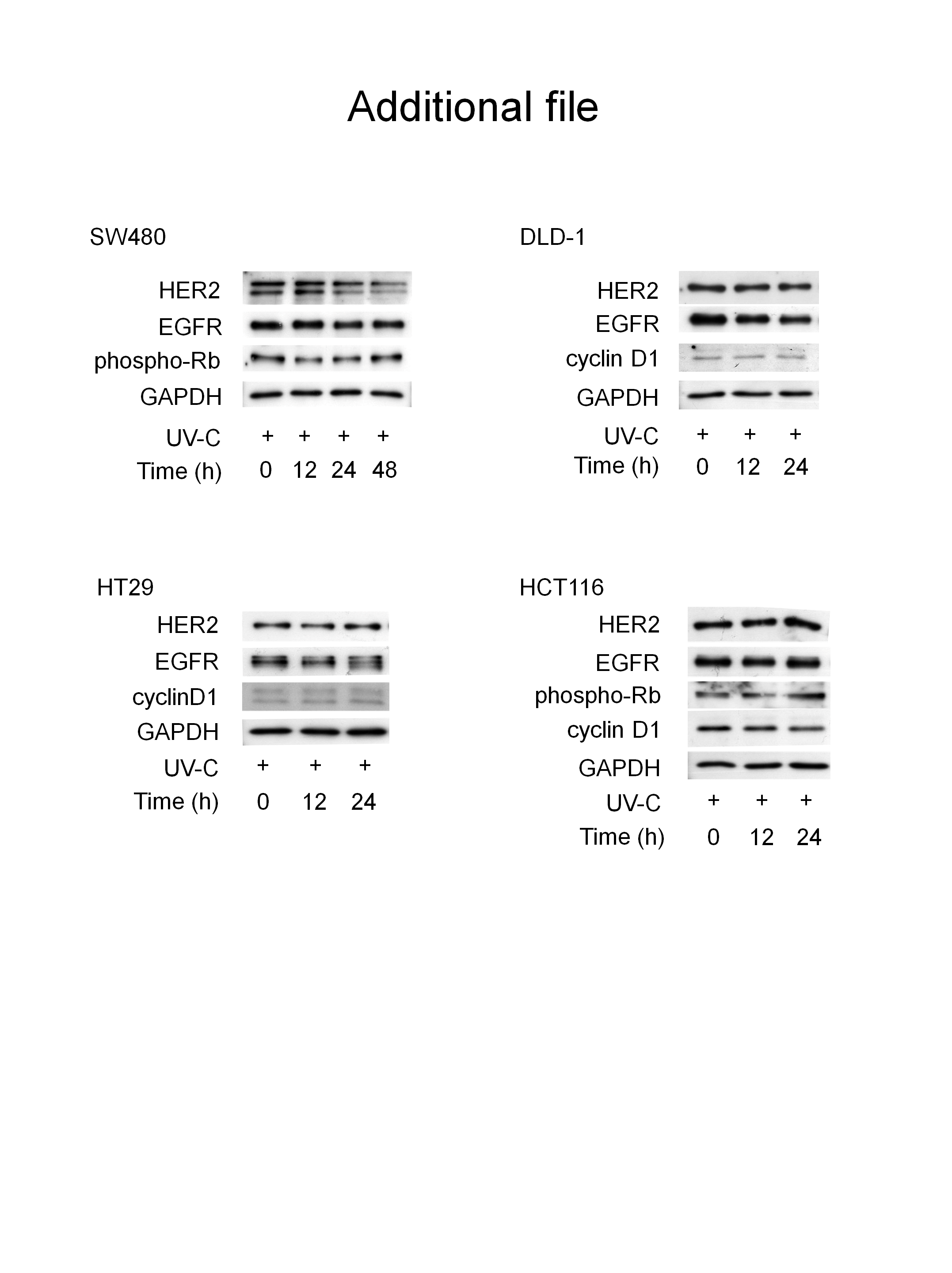

Supplement: Additional file 1 — Effect of 10 J/m² UV-C on HER2, EGFR, phospho-Rb and cyclin D1 in human colorectal cancer cells. SW480, DLD-1, HT29 and HCT116 cells were exposed to 10 J/m² of UV-C and then treated for the indicated periods. Protein extracts were then harvested and examine by Western blotting using anti-HER2, anti-EGFR, anti-phospho-Rb, anti-cyclin D1 and anti-GAPDH antibodies. [file 1476-4598-11-45-S1.tiff]
